# Danish primary care: a focus on general practice in the Danish healthcare system

**DOI:** 10.1080/02813432.2025.2508929

**Published:** 2025-05-22

**Authors:** Peter Haastrup, Anne Møller, Jette Kolding Kristensen, Linda Huibers

**Affiliations:** ^a^ Research Unit of General Practice, Department of Public Health, University of Southern Denmark, Odense, Denmark; ^b^ The Research Unit for General Practice and Section of General Practice, Department of Public Health, University of Copenhagen, Copenhagen, Denmark; ^c^ Center for General Practice at, Aalborg University, Aalborg, Denmark; ^d^ Research Unit for General Practice, Aarhus, and Department of Public Health, Aarhus University, Aarhus, Denmark

**Keywords:** Denmark, general practice, primary care, organization

## Abstract

Denmark is known for its good population health, largely attributable to its effective healthcare system. This analysis of the Danish primary healthcare system with focus on general practice describes the system’s overall structure, function, and financing. Further, it reviews some of the recent developments in organization and decentralization from secondary to primary care. Finally, we discuss some of the key challenges that primary care faces and potential areas for improvement to ensure a sustainable Danish healthcare system of high quality.

## Introduction

The Danish healthcare system is often regarded as a model of efficiency and equity due to its equal access for all residents, its emphasis on preventive care, and its striving to solve healthcare issues at the lowest cost level. Funded primarily through taxes, the system provides healthcare services at little or no cost to the patient, reducing the financial barriers often associated with medical care [1]. Among its many components, primary care stands out as the cornerstone of the system. General practitioners (GPs) are gatekeepers of specialized services, ensuring that patients receive appropriate and coordinated care. Since the last description by *Pedersen* et al. in 2012 [2], the Danish healthcare system has gone through several changes and reforms. Therefore, we aim to provide an updated description of the Danish healthcare system, emphasizing the role of primary care and general practice. As such, we describe the structure, function, and financing of the system, and the position of primary care in the broader healthcare landscape. Moreover, we explore the key challenges that primary care faces and potential areas for improvement.

## The Danish healthcare system: an overview

The Danish healthcare system is divided into three administrative levels: the state, five regions and 98 municipalities. The system is decentralized, and each level is financed by separate taxes. Planning, administration and regulation take place at all three levels. The state holds the overall regulatory, supervisory and fiscal functions, and public healthcare legislation constitutes the framework for the authorities’ administration of healthcare tasks. The regions are responsible for hospitals and paying for primary care services, mostly delivered by self-employed healthcare professionals. The municipalities handle preventive healthcare, addictive diseases, child dental service, health visitors, rehabilitation and long-term care for the elderly and disabled, both in nursing homes and the citizens’ homes. Further, the municipalities have several tasks other than healthcare, for example, social care, unemployment management, childcare, public schools, etc.

At all levels, the healthcare system is managed by a combination of democratically elected politicians and professional civil servants. This decentralized approach allows for regionally tailored healthcare services, while the central government provides the overall direction and control.

## Financing

The healthcare system is largely funded by taxes. Patients face minimal out-of-pocket expenses, mostly for services like adult dental care, drugs, psychologists, and physiotherapy/chiropractors that are all partly self-paid. Expenses for prescription drugs are subsidized, so the more expenses a citizen has for reimbursable medicine, the more reimbursement is received within one year (the reimbursement period) [3]. This limits the expenditures of each citizen for prescription medicine. Further, supplementary individual reimbursement can be applied in case of financial need or terminal disease.

The tax-funded nature of the healthcare system ensures that all citizens have access to primary care services, regardless of income, insurance, or employment status. In 2022, the total regional expenses for general practice services were 10.5 billion DKK, corresponding to an average annual cost of 1,540 DKK (207 €) per citizen [4]. The total Danish healthcare budget was 266 billion DKK in 2022, where regional expenses for hospitals were 116.3 billion DKK, municipal expenses for elderly care, long-term care/nursing homes, aids, and transportation were 59.9 billion DKK, and expenses for dental care were 13.3 billion DKK. Drug reimbursement expenses were 7.6 billion DKK in 2024.

## Primary care in Denmark

### Structure and role of primary care

Primary care in Denmark is largely delivered by GPs, who serve as the first point of contact for patients and are responsible for managing most medical conditions. GPs act as gatekeepers, determining whether a patient needs a referral for specialized services. This gatekeeping function is central to the Danish healthcare system and ensures that patients are treated at the appropriate level of care, reducing unnecessary use of specialist services and hospital admissions [5]. A total of 87% of the population contact their GP each year [6]. Approximately 90% of these 44 million contacts are managed in primary care without further referral.

General practice operates a list-based system, meaning that 99.5% of the citizens are registered with a specific GP. This supports a long-term relationship between GP and patient and thus fosters continuity of care. Continuity is crucial for managing chronic diseases and coordinating care in various healthcare settings [7,8]. The trusting relationship between GP and patient is developed during repeated consultations about various topics. Subsequently, communication can be more effective and tailored. Furthermore, GPs can practice individualized medicine, noticing and reacting to subtle changes in a patient’s health status, which increases timely diagnosis [9,10]. On average, a GP has 1,700 patients on the list.

However, citizens can decline to be listed with a specific GP (about 0.5% of the population). They will have to pay a fee for primary care services but are free to contact any GP and consult any private practicing specialist directly without being referred by a GP. Patients on the list need a referral for all other specialties, except private practicing ophthalmologists and otorhinolaryngologists.

To receive reimbursement from the region for listed patients, GPs need a provider number. The health authorities control the distribution of these provider numbers and thereby the supply of GPs in different areas. GPs can sell their provider number and clinic facilities whenever they wish, in transactions like any other business, although restricted by several rules. GPs are remunerated by a mixture of per-capita fees and fee-for-services. The per-capita fees are to a small extent differentiated so that a higher fee is given to GPs working in areas with few GPs and/or with a list of patients with high age or high degree of comorbidity [11]. Approximately two-thirds of the GP’s income comes from fee-for-service payments and one-third comes from capitation payments for patients on the GP’s list.

Danish GPs are organized in the Danish Organization of General Practitioners (“*PLO*”), which is a subdivision of the Danish Medical Association. This organization negotiates a collective agreement on financing, obligations, and privileges of GPs with the Danish regions. The agreement is renegotiated with a few years’ interval. This allows ongoing adjustments of GP tasks, remuneration, and incentive schemes to increase GPs’ attention to certain tasks. As an example, the comparatively high fee for preventive consultations among patients with severe psychiatric disorders is supposed to increase the GPs’ attention to this patient group and encourage GPs to offer longer consultations that focus on broader health and prevention activities among these patients, where increased allocated time can be necessary to cover both mental and somatic health [12].

The collective agreement states that GPs are obliged to offer consultations through telephone, e-mail, face-to-face and digitally through secure video access. The GP should be available all working days from 8 AM to 4 PM and at least one day a week later than 4 AM. In case of a holiday or other absence, the GP is responsible for arranging a named substitute GP for all patients.

Any conflicts or disagreements that may arise regarding the collective agreement are usually solved by consensus in the regional or national board of collaboration with representatives from both the regions and the GPs.

Most GPs are also members of the Danish College of General Practitioners (“*DSAM*”), which is the professional and scientific society of GPs. The Danish College of General Practitioners focuses on education, international cooperation, research and quality in general practice. For example, the college develops and distributes guidelines relevant and adjusted to general practice regarding topics such as the management of diabetes, osteoporosis, dyspepsia, out-of-hours triage, etc. The college is a part of the Nordic Federation of General Practice and, in collaboration with the other Nordic colleges, arranges the biannual Nordic Congress of General Practice.

## Organization of general practice

GPs are organized in solo or group practices, with most GPs working in group practices that can be either GPs with separate patient lists cooperating with shared clinic premises, staff, laboratory equipment, etc., or partnership practices with a shared patient list, where the patient is listed with the entire clinic instead of a specific GP.

GPs are distributed locally and despite regional differences, the average distance from a citizen’s home to the GP is about 5 kilometers. Thus, transportation is less of a barrier for GP contact compared to other countries with greater geographical distances. However, there is a substantial variation in the distance from the citizen’s home to the GP among regions and, in some areas, the distance can be a barrier [13]. Pensioners without possibilities of transportation to the GP, specialist, hospital, or rehabilitation can apply for municipal transportation and/or reimbursement and an accompanying person if needed. Among the elderly, who often keep their addresses, only a few citizens change their general practice [14]. A high level of continuity is thus provided, which is associated with lower use of healthcare services, lower risk of hospitalization, and lower mortality [9,10].

GPs work as independent contractors and are responsible for the economy, equipment, and organization of their practice. All general practices are fully computerized. Various software systems are available to handle patient records, send electronic prescriptions to pharmacies and referrals to specialists or hospitals, communicate with the municipalities, and receive laboratory analysis results and hospital discharge letters. GP practices have point-of-care tests available, such as hemoglobin, C-reactive protein, blood glucose, urine dipsticks and microscopy, ECG, streptococcal antigen test, etc. A growing number of practices also has ultrasound [15].

GP’s tasks range from maternity care, prevention of diseases, handling of newly arisen problems, and managing known conditions or diseases among patients of all ages, including pediatric and geriatric issues. Traditionally, GPs have taken care of their patients even after they moved to a nursing home, which resulted in many GPs visiting the same nursing home and fragmentation of care. Nowadays, most nursing homes have a specific and dedicated GP affiliated, who is available for questions regarding the nursing home residents and who regularly visits the residents for consultations and education of the staff [16]. A similar arrangement is planned for homes for people with intellectual/physical disabilities from 2025.

Being independent contractors, GPs can freely decide how to structure their daily clinical work as long as the collective agreement is kept. This means that the tasks of a GP are standardized, but that the execution of these tasks can be very diverse. Further, being independent contractors means that the individual GP organization can be very agile and able to implement organizational changes rapidly.

Danish GPs employ a range of healthcare professionals, including secretaries, nurses, medical students, etc. Medical students are increasingly been used to perform secretarial and nursing tasks. Consequently, the number of staff employed in general practice has been increasing over the past decade, and management and other employer tasks are important parts of the everyday life of Danish GPs.

For decades, out-of-hours primary care was delivered in large-scale GP cooperatives. These GP cooperatives provided care outside the GP’s office hours, from 4 pm to 8 am on weekdays, weekends, and holidays [17]. These GP cooperatives were organized by the regional Organizations of General Practitioners and consisted solely of GPs. However, in all regions, the out-of-hours primary care service has been reorganized due to high demand, lack of workforce, and demographic changes. The regions have the formal responsibility for providing primary care, with the GPs having the obligation to provide 24/7 patient care; hence, different regional organizational models currently exist within the same national context [18].

Patients must contact the out-of-hours primary care service by telephone. In these GP cooperatives, so-called triage GPs answer the call and perform telephone triage to assess the urgency of the patient’s problem and the level of care needed. GPs can use one-way video in their telephone contacts [19]. The triage GP can end the call by telephone or refer to a face-to-face contact with a GP. A telephone consultation can end with providing self-care advice, prescribing medication, and/or referring to the patient’s own GP, or referring directly to the hospital (if needed with the dispatch of an ambulance). At the out-of-hours clinic, GPs perform clinic consultations. These clinics are mainly located at or near a hospital, which supports the options of task sharing. GPs have access to a limited range of diagnostics and nurse support. GPs also provide home visits, supported by a driver with limited training and medical equipment available.

In 2014, the Capital Region of Denmark decided to take home the organization of primary care outside office hours from the GPs. They established a medical helpline called 1813, which can be accessed 24/7 by telephone [20]. Here, nurses perform telephone triage, supported by a computerized decision support system. A range of physicians is also employed to supervise telephone triage, provide support, and perform telephone triage. The medical helpline seldom provides home visits; a patient in need of face-to-face contact is referred to a nearby hospital. Here, patients are seen by a physician (infrequently a GP).

In 2022, Region Zealand also took back the responsibility for primary care outside office hours from the regional Danish Organization of General Practitioners. Here, GPs and other doctors work as triage doctors, and they have the same possibilities as in the three other regions (i.e. the Central Denmark Region, the Northern Denmark Region, and the Southern Denmark Region). These last three regions also changed their organization in 2024, after a collaborative process between the region and the Organization of General Practitioners. Here, the existing GP cooperative continues unchanged from 4 am to 11 pm on weekdays, and from 8 am to 11 pm on weekends and holidays. After 11 pm, the emergency medical service (ambulance care (112)) is responsible for the provision of primary care in the Central Denmark Region and the Northern Denmark Region, and the hospital emergency department in the Region of Southern Denmark, with different organization of telephone triage and home visits.

## Education in general practice

After six years in medical school and graduation, at least six years of postgraduate training is required to become a GP in Denmark. Postgraduate training includes one-year basic clinical medical training (compulsory for all doctors irrespective of subsequent specialty) followed by five years of standardized GP training, including employment in two or three different GP clinics, and hospital employment at departments of internal/emergency medicine, gynecology and obstetrics, pediatrics, psychiatry, and general surgery. During vocational GP training, the doctors also follow a theoretical course program and research training. The curriculum is competency-based according to the CanMEDS professional roles, comprising biomedical knowledge, leadership, etc [21].

There are currently approximately 3,500 GPs in Denmark and 1,200 doctors in specialist training to become GPs. In 2024, a total of 265 doctors completed GP specialist training. In comparison the total number of specialist doctors (all medical specialties) in Denmark is approximately 17,500.

After vocational training, the doctor receives the title of Specialist in General Medicine and can buy a provider number and run a GP clinic. There is no requirement for recertification, but in the collective agreement between the GPs and the regions, funds for remunerated continuing educational activities are allocated for each GP [22] and some courses are mandatory. Each GP can apply for an annual continuous medical education refund of approximately €6,500 per year. Despite the well-remunerated and comprehensive continuous medical education model, one-quarter of Danish GPs did not use remuneration for continuous medical education in 2022, often due to time restraints, fully booked courses, or difficulties finding a substitute doctor [23].

## Research in general practice

The collective agreement between the regions and the GPs secures an annual amount for research administered by the Danish Research Foundation of General Practice. Research units have been established at each university in the four largest cities and are largely co-funded by external grants. Each research unit investigates and teaches various aspects of general practice and is headed by a GP-professor. The research units have a high degree of collaboration, but each unit employs its own scientific and administrative staff. The scientific employees include senior researchers, PhD students, MSc students completing a research year, etc. The research units employ a multidisciplinary scientific staff that includes GPs or GP trainees, as well as other health professionals, masters in public health, statisticians, anthropologists, psychologists, etc. The scientific staff is often part-time employed at the university/research unit and part-time in general practice. The number of academic staff largely depends on external funding, Each year the research units publish a substantial number of academic papers contributing to international academic general practice. A total of 4–17 PhD theses are defended at the research units each year. In 2024, a total of 375 publications derived from the four research units.

## Preventive care and health promotion

Preventive care is a key component of the Danish primary care system. GPs provide family-centered preventive check-ups for children and pregnant women (including vaccinations). Each pregnant woman is offered a minimum of three preventive check-ups at the GP office during the pregnancy, supplemented by an ultrasound screening program at the hospital to detect fetal malformations. as well as ambulatory midwife check-ups. After the child is born, the family is offered a postpartum check-up by their GP for the mother, and regular preventive check-ups for the child at the ages of five weeks, five months, and thereafter around their birthday until the age of five [24].

GPs provide the pap smear screening program, but other screening programs are led by the regions (i.e. colorectal cancer and mammography), and GPs are not involved in these programs.

Besides preventive family care, GPs offer all relevant patients counseling on lifestyle changes, management of chronic diseases, and risk factors such as obesity, smoking, and hypertension. Preventive care initiatives are further supported by the municipalities, which offer services such as public health campaigns, smoking, alcohol, and substance abuse cessation programs, and exercise classes for at-risk populations.

## Integration with secondary care

The primary care system in Denmark is closely integrated with secondary and tertiary care services. GPs collaborate with specialists and hospitals to ensure coordinated and timely care for patients requiring more complex investigations or treatments. Referrals from GPs are required to access specialist care, thereby ensuring that patients are treated within the primary care setting whenever possible. Further, hospitals send discharge letters to the GP. This integration aims to reduce fragmentation of care and support the continuity of treatment across different healthcare providers. However, communication is often unidirectional where close cross-sectoral communication and collaboration regarding single patients still is under development [25,26]. Further, rejection of referrals is a problem hindering optimal cooperation and causing frustration [27].

## Challenges facing primary care in Denmark

Despite the many strengths of the Danish primary care system, it faces several challenges as suggested by OECD [28,29]. Although Denmark has a high score in OECD indicators like vaccination coverage and citizens’ satisfaction with the availability of quality healthcare services, there is still room for improvement in other OECD indicators, such as mortality rate from preventable/treatable causes compared to other Western countries [29].

The aging population and consequent increasing number of complex patients with multimorbidity [28], increasing demand for healthcare services, and task delegation from secondary to primary care have put significant pressure on the primary care workforce. In many areas, shortages of GPs have led to increased workloads for existing GPs, which potentially complicates the accessibility for patients, job satisfaction, and mental well-being of GPs [30]. Despite being a relatively small country, there is substantial geographical variation in health in Denmark. The prevalence of obesity, daily smoking, and chronic disease is higher in deprived or remote areas than in more advantaged or urban areas [28]. The paradox that the number of doctors often is lower in areas with high deprivation compared to areas with no deprivation (the so-called *inverse care law* [31]) constitutes a vicious circle where GPs with a deprived patient population may experience a high work pressure, have insufficient time for comprehensive tasks and are at higher risk for developing burnout [32].

Efforts are being made to recruit more medical professionals in general practice, but attracting young doctors to rural or deprived areas remains a challenge. Due to the shortage of GPs in some regions, private companies or regional practices are taking over the management of vacant GP clinics. In such clinics, the employed physicians are often temporary, which hinders continuity. Currently, 15 GP clinics are run by the regions.

The rising prevalence of chronic diseases with multiple treatment options, such as diabetes and cardiovascular disease, has increased the demand for long-term management within the primary care setting. While Denmark’s primary care system is experienced in managing chronic conditions, the increasing burden of these diseases may require additional resources and restructuring to ensure that care remains efficient and high-quality. In many general practices, tasks have been delegated from the GP to other healthcare professionals e.g. nurses, who are often responsible for the management of diabetes, hypertension, COPD, etc. This task delegation allows the GP to focus on other diseases and diagnostics, but the downside may be hampered GP-patient continuity [33].

Denmark has traditionally had a high degree of work satisfaction among GPs compared to other European countries [34]. To maintain a high degree of work satisfaction, health policymakers need to keep in mind that diversity of work, relations and contact with colleagues, and being involved in teaching medical students are factors known to increase job satisfaction among GPs, while low income, too many working hours, administrative burdens, heavy workload, lack of time, and lack of recognition are factors negatively affecting the job satisfaction of GPs [35].

## Conclusion

In the Danish primary healthcare system general practice plays an important role in ensuring accessibility, continuity, and cost-effectiveness in healthcare delivery. Denmark’s primary care system is considered one of the most efficient and equitable in the world [36], but substantial challenges are posed by workforce shortages and the increasing burden of chronic diseases, so a continuous effort to secure GPs’ well-being and job satisfaction is needed.

Efforts to strengthen primary care, including investment in the workforce, better integration of care, and the continued emphasis on preventive services are planned and a new political healthcare reform will be fully implemented in 2027 [37]. Such continuous effects and focus will be crucial in ensuring a sustainable system that continues to meet the evolving needs of the Danish population. Maintaining a strong focus on primary care and general practice is essential to address future healthcare challenges and secure high-quality, accessible care to all citizens.
